# Meta-Analysis of the Effect of Kangfuxin Liquid on Diabetic Patients with Skin Ulcers

**DOI:** 10.1155/2021/1334255

**Published:** 2021-06-03

**Authors:** Xiaoping Wan, Funeng Gen, Yongmei Sheng, Meng Ou, Fang Wang, Ting Peng, Jinlin Guo

**Affiliations:** ^1^Key Laboratory of Systematic Research of Distinctive Chinese Medicine Resources in Southwest China, Chengdu University of Traditional Chinese Medicine, Chengdu, Sichuan 611137, China; ^2^Innovative Institute of Chinese Medicine and Pharmacy, Chengdu University of Traditional Chinese Medicine, Chengdu 611137, China; ^3^Sichuan Key Laboratory of Medical American Cockroach, Chengdu 611137, China

## Abstract

In this study, we used meta-analysis to comprehensively evaluate the clinical efficacy of Kangfuxin Liquid in the treatment of diabetic patients with skin ulcers. Literature search was performed through PubMed, Web of Science, Embase, China National Knowledge Infrastructure, and Wanfang Data. The retrieval was not limited by language, and the search period was from 2010 to October 12, 2020. Diabetic patients with skin ulcers were treated with Kangfuxin Liquid combined with basic treatment as the treatment group and only basic treatment as the control group. Stata16.0 software was used for system evaluation. A total of 11 studies and 874 patients were included. Meta-analysis showed that 11 studies compared the treatment efficacy between the two groups, and the results showed that the treatment efficacy in the treatment group was significantly higher than that in the control group [OR = 5.38, 95% CI (3.52, 8.24), *P* < 0.001]. Among them, 9 studies compared the healing time of wounds. The healing time of the treatment group was significantly longer than that of the control group [SMD = −2.13, 95% CI (−2.85, −1.41), *P* < 0.001]. Five studies compared the length of stay, and the length of stay in the treatment group was shorter than that in the control group [SMD = −3.68, 95% CI (−5.38, −1.97), *P* < 0.001]. Compared with basic treatment, Kangfuxin Liquid combined with basic treatment has an ideal effect in the treatment of diabetic skin ulcers, which can improve the overall treatment efficiency and shorten the wound rehabilitation time and the length of stay.

## 1. Introduction

The main cause of chronic skin ulcers is diabetes or bacterial infection, followed by pressure, traumatic ulcer, and anti-inflammatory ulcer. In recent years, the prevalence of chronic skin ulcers has increased, which may lead to soaring mortality [1]. Diabetes mellitus is a widespread chronic endocrine disease [2]. According to statistics, 8.4% of the global disease-related deaths are caused by diabetes mellitus in adults aged 20–79 years. Moreover, there are nearly 5.1 million deaths. The estimated attributed mortality is between 5.1% (3.3 million deaths) of the total mortality and 10.1% (6.6 million deaths) of the total mortality [3]. Diabetic complications are often an important cause of disability or death in diabetic patients, with 30% of diabetic patients experiencing skin changes that often occur before diagnosis, for example, experiencing infectious skin diseases, such as fungal and bacterial infections, and noninfectious diseases, such as itching, lipid necrosis, and granuloma annulare [1]. Once diabetic ulcers occur, it is difficult for people to treat them, and a long period and a high cost are also required for treatment, which greatly affects the life quality of patients [4]. In view of the symptoms of diabetic skin ulcers, the common clinical treatment methods mainly include blood glucose control + debridement and dressing change therapy [5]. However, there is no complete consensus on the treatment methods and efficacy of skin ulcers in previous studies.

Kangfuxin Liquid is a solution made from extracts of *Periplaneta americana* and is rich in active substances such as peptides, polyols, and sticky sugar amino acid (SSAA) [6]. Studies have shown the role of polyphenol-rich compounds in health and disease. Curcumin can effectively reduce oxidative stress-related complications in patients with polycystic ovarian syndrome, and ginger can decrease the circulating CRP, hs-CRP, and TNF-*α* levels [7, 8]. In addition, pomegranate has a positive effect on oxidative stress parameters [9]. It provides a reference for the research of Kangfuxin Liquid. It is commonly used in clinical practice to treat various ulcerative diseases [10, 11]. It promotes the proliferation of immunocompetent cells such as neutrophils, promotes neovascularization, improves microcirculation, accelerates the proliferation of granulation tissue, and allows the body to rapidly repair injured tissues, thereby reducing ulcer healing time [12]. Diabetes mellitus complicated with skin ulcers is common in clinical practice, which often brings great pain to patients. In China, Kangfuxin Liquid has been used clinically for diabetic foot ulcers [13]. However, there is no systematic review to evaluate the efficacy of Kangfuxin Liquid in the treatment of diabetic skin ulcers. The aim of this systematic review and meta-analysis of published randomized controlled trials (RCTs) is to evaluate the clinical effects of Kangfuxin Liquid on the treatment of diabetes skin ulcers. We comprehensively and quantitatively compared the advantages and disadvantages of both Kangfuxin Liquid combined with basic treatment and basic treatment on the clinical efficacy of diabetic skin ulcers and explored the optimal treatment for diabetic skin ulcers.

## 2. Materials and Methods

### 2.1. Literature Retrieval

English databases such as PubMed, Web of Science, and Embase were searched with the English search terms (“Kangfuxin Liquid” OR “KangFuXin solution” OR “Periplaneta americana”) AND (“diabetes” OR “diabetes mellitus”) AND (“Skin ulcer”). Chinese databases such as China National Knowledge Infrastructure and Wanfang Data were searched with the combination of the Chinese search terms: “Kangfuxin Liquid, diabetes, and skin ulcer”. Meanwhile, possibly related literature was selected through the second retrieval of relevant literature references and conference papers, abstract articles, etc. The retrieval was not limited by language, and the retrieval time was from 2010 to October 12, 2020.

### 2.2. Screening Criteria

Inclusion criteria were as follows. (1) Patients who met the diagnostic criteria of diabetic skin ulcers were included. (2) Kangfuxin Liquid was used as the main treatment. The treatment group was treated with Kangfuxin Liquid combined with basic treatment, whereas the control group only received basic treatment. Basic treatment included controlling blood glucose, anti-infection, and routine debridement. (3) The main outcome was the total effective rate of treatment. Judgment criteria were as follows: remarkably effective, wound healing after treatment, new skin formation, and no wound exudation; effective, after treatment, partial wound healing, with a small amount of granulation tissue and a small amount of exudation; ineffective, failure of wound healing, increased exudation, and wound enlargement. Wound healing time and hospitalization time were also evaluated.

Exclusion criteria were as follows: (1) articles not related to diabetic skin ulcers, poor quality of literature, missing data, and repeated publications; (2) studies where statistical methods are not rigorous or operational processes are inappropriate; (3) nonclinical studies such as case reports, systematic reviews, and theoretical researches.

### 2.3. Literature Quality Assessment and Data Extraction

The literature was independently screened by two investigators according to the inclusion criteria, and the quality, characteristics, and results of the data were abstracted. Disagreements regarding data extraction were resolved by discussion and consultation with a third researcher. Data extraction includes authors, publication year, the sample size of treatment group and control group, intervention measures, observation indicators, and results.

### 2.4. Statistical Analysis

Stata16.0 software was used for input and analysis. Heterogeneity of the included studies was assessed using the Cochran's Q test and the *I*^2^ statistic, and the random-effects model was used to combine the statistics for the result data with high heterogeneity (when *P* < 0.05 and *I*^2^ > 50%); otherwise, the fixed-effects model was used. Continuous data were analyzed for the primary outcome data by calculating the overall mean difference of the study for the standardized mean difference (SMD) and its 95% confidence interval (CI). Odds ratio (OR) and its 95% confidence interval (CI) were calculated for categorical variables. *P* values < 0.05 were considered statistically significant. Sensitivity analysis was used to evaluate the degree of robustness and credibility of the results of the meta-analysis. Funnel plots, Begg's test, and Egger's test were used to evaluate whether the results had potential publication bias, and the test level *α* was taken as 0.10.

## 3. Results

### 3.1. Status of Included Literature

**Figure 1:**
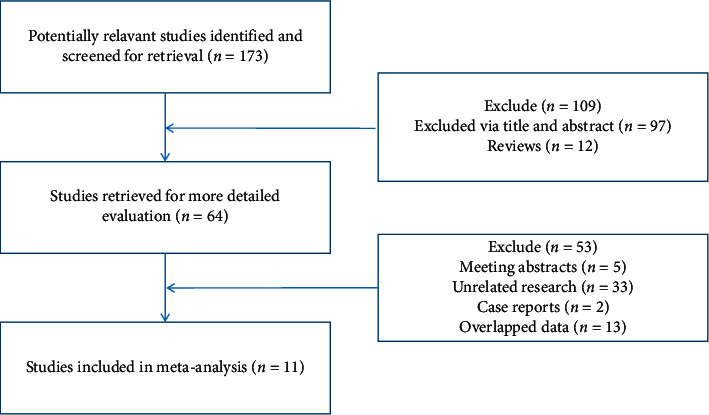
Flowchart of literature screening.

A total of 173 articles were retrieved: 109 articles inconsistent with the inclusion and labeling were excluded according to the title, abstract, and full text, and 64 articles were selected. Further, 53 articles with conference abstracts, case reports, and duplicated data were removed; finally, 11 studies met the selection criteria [5, 14–23], all of which were Chinese literature. There were a total of 874 patients, including 440 patients as the treatment group in the trial of Kangfuxin Liquid combined with basic treatment and 434 patients in the control group. The screening process of the literature is shown in Figure 1, and the characteristics of the included literature are shown in Table 1.

### 3.2. Results of Meta-Analysis

#### 3.2.1. Treatment Efficiency

**Figure 2:**
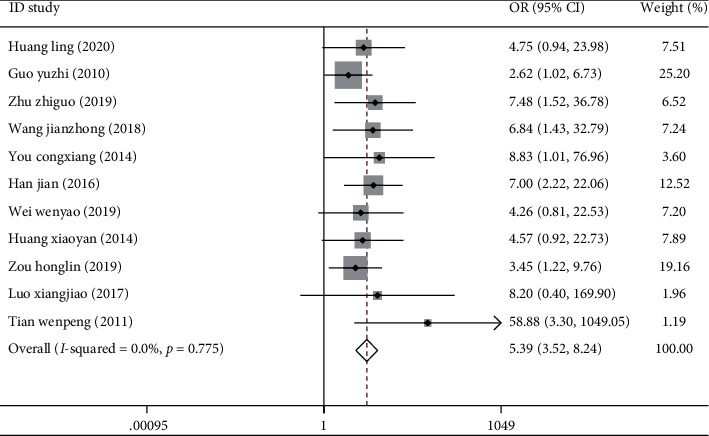
Forest map of treatment response rate in two groups of diabetic patients with skin ulcers.

All 11 studies discussed the effective rate of Kangfuxin Liquid in the treatment of skin ulcers. The data combined showed that the heterogeneity of the two indicators of each study was large (*I*^2^ = 0.00%, *P*=0.775), so the random effect model was used. Meta-analysis showed that the effective rate after treatment in the treatment group was significantly higher than that in the control group, 5.38 times that in the control group [OR = 5.38, 95% CI (3.52, 8.24), *P* < 0.001] (Figure 2).

#### 3.2.2. Wound Healing Time and Hospitalization Time

**Figure 3:**
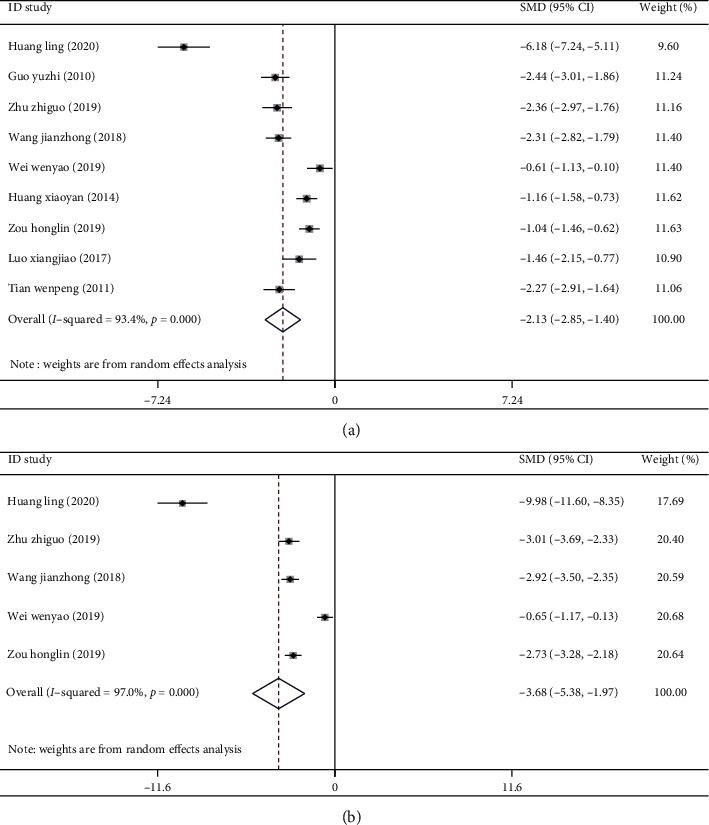
Forest map of wound healing time and hospitalization time of patients with diabetic skin ulcers in two groups: (a) Forest map of wound healing time of patients with diabetic skin ulcer. (b) Forest map of hospitalization time of patients with diabetic skin ulcer.

There were 9 studies [5, 14–16, 19–23] where the outcome variable was wound healing time. As shown in Figure 3(a), the heterogeneity of the two indexes in each study was large (*I*^2^ = 93.40%, *P* < 0.001), so the random effect model was used. Meta-analysis showed that Kangfuxin Liquid combined with basic treatment could significantly shorten the wound healing time of patients with skin ulcers, and the difference had statistical significance [SMD = −2.13, 95%CI(−2.85, −1.41), *P* < 0.001].

The outcome variable of 5 studies [5, 15, 16, 19, 21] was the hospitalization time of patients. The merging of the data showed that the heterogeneity of the two indexes in each study was large (*I*^2^ = 97.00%, *P* < 0.001), so the random effect model was adopted. The results of meta-analysis showed that the hospitalization time of the treatment group was significantly shorter than that of the control group, and the difference was statistically significant [SMD = −3.68, 95%CI(−5.38, −1.97), *P* < 0.001] (Figure 3(b)).

#### 3.2.3. Sensitivity Analysis

**Figure 4:**
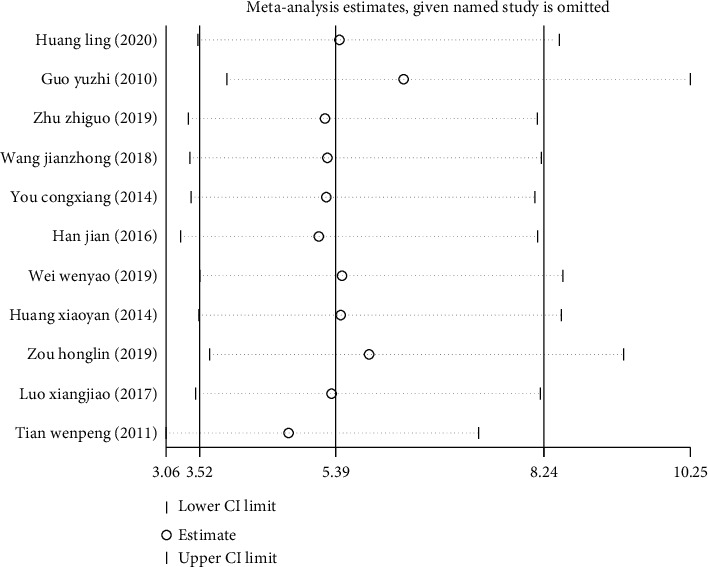
Sensitivity analysis of treatment efficiency of two groups of patients with diabetic skin ulcers.

**Figure 5:**
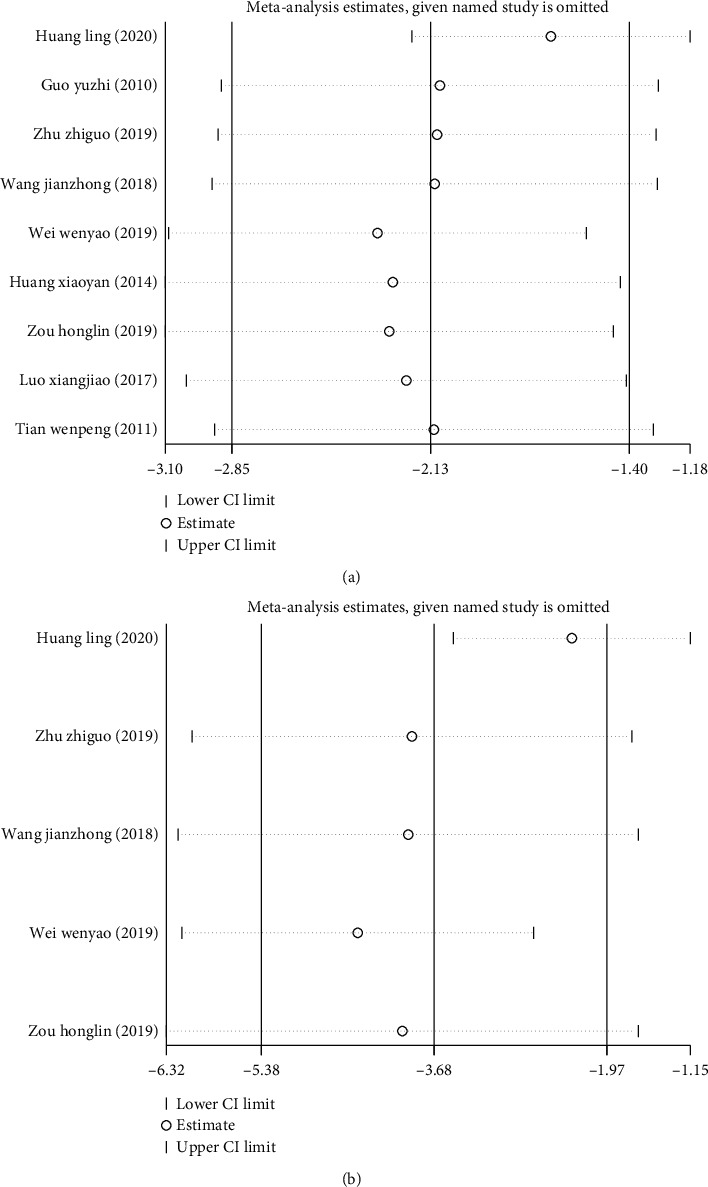
Sensitivity analysis of wound healing time and hospital stay in patients with diabetic skin ulcer. (a) Sensitivity analysis of wound healing time in patients with diabetic skin ulcer. (b) Sensitivity analysis of hospital stay in patients with diabetic skin ulcer.

Sensitivity analysis showed that the combined results of treatment efficiency (Figure 4), wound healing time (Figure 5(a)), and hospitalization time (Figure 5(b)) did not change in the included studies, which confirmed that the results of this meta-analysis were robust and credible.

#### 3.2.4. Publication Bias

**Figure 6:**
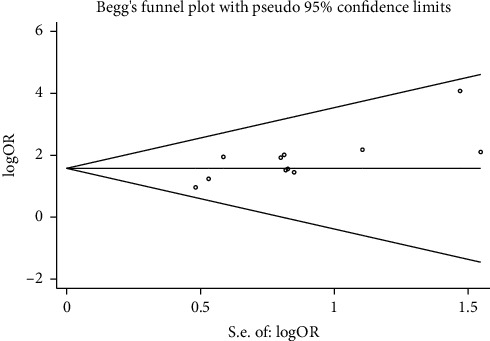
Funnel plot of treatment efficiency of two groups of patients with diabetic skin ulcers.

Funnel plots were used to detect possible publication bias. The results of Begg's test (*Z* = 1.09, *P*=0.276) and Egger's test (*t* = 0.94, *P*=0.373) showed that there was no publication bias in the treatment efficiency between the two groups (Figure 6). Since the publication bias exists by default in the number of articles below 10 in the analysis with wound healing time and hospitalization time as outcome variables, there was no funnel plot analysis to perform.

## 4. Discussion

Diabetes is a chronic disease caused by long-term glucose metabolism disorder. The pathogenesis is complex, and it is often accompanied by multiple systemic organ lesions. Skin lesions are one of the important complications of diabetes [24]. Some studies have also found that neuropathy and vascular lesions caused by diabetes may lead to skin lesions [1]. Long-term diabetes can cause vascular lesions, angiostenosis, and angiosclerosis, affect the blood supply of patients' skin, and lead to nutritional disorders of the skin. Metabolic disorders not only cause nerve damage but also affect the ability of nerve self-repair. Nerve and neuropeptides play an important role in the normal immune function and tissue repair of the skin. Once tissue repair is poor or infection occurs, skin ulcers exist [25]. Previous studies have found that fibroblasts are the main repair cells in the healing of diabetic skin ulcers. They participate in collagen synthesis and secretion, affect the synthesis of extracellular matrix, and control proliferation, differentiation, and metabolism of cells [26]. In summary, skin lesions and vascular lesions caused by diabetes are the basic causes of skin ulcers. The course of treatment of skin ulcers is long; the prognosis is poor; the healing time is long. If early intervention cannot be obtained, severe cases may lead to diabetic foot, lower limb tissue structure, or skin tissue damage and ultimately often lead to amputation or toe amputation. Long-term hyperglycemia increases the sugar content in the exudation of skin ulcers, which causes bacterial reproductive infection and increases the possibility of infection and slow healing [15]. Due to the high incidence, complex treatment, and slow healing process of diabetic skin ulcers, clinical treatment is generally based on improving vascular lesions and protecting nerves, and surgical treatments apply debridement, dressing change, or other special dressings. However, the therapeutic effect is different [27, 28].

This meta-analysis compared the effect of Kangfuxin Liquid combined with basic treatment and basic treatment in the clinical treatment of patients with diabetic skin ulcers. Through the comparative results of meta-analysis, it is found that the former has more prominent therapeutic advantages, which is first reflected in the efficiency of Kangfuxin Liquid combined with a basic treatment for diabetic skin ulcers compared with basic treatment. Kangfuxin Liquid is a commonly used clinical drug for the treatment of diabetic skin ulcers in clinical practice. The use of Kangfuxin Liquid combined with basic treatment can obtain a higher treatment efficiency. Studies have found that wet compress treatment with Kangfuxin Liquid can promote the proliferation of neutrophils, macrophages, and lymphocytes in the wound, improve spontaneous and chemotactic function [29], promote the shedding of necrotic tissue, promote the growth of new granulation tissue, and promote tissue repair [30]; at the same time, it can upregulate the level of glutamine, stimulate protein secretion, improve wound microcirculation, and accelerate the proliferation of fibroblasts [31].

Nine of the included studies compared wound healing time. The results showed that Kangfuxin Liquid combined with basic treatment could significantly shorten the wound healing time of patients with skin ulcers. There were five studies that compared patients' hospitalization time. The results showed that the hospitalization time in the test group was significantly longer than that in the control group. The wound healing time and hospitalization time of patients with Kangfuxin Liquid combined with basic treatment were shorter, indicating that performing this method for the treatment of diabetic skin ulcers is more conducive to the rehabilitation of patients' condition. Meanwhile, patients can be discharged as soon as possible and reduce the physical and mental burden and economic pressure of patients. At the same time, attention should be paid to early intervention of diabetic skin ulcers to improve the prognosis, avoid the continued development of ulcers into deep refractory ulcers, reduce the incidence of sequelae, and reduce the health damage caused by skin ulcers.

No doubt, this meta-analysis also has limitations. First, due to the retrospective study and small study sample size, there may be some selection bias and information bias. Second, due to the limited relevant information provided in the literature, the effects of various confounding factors such as age, gender, diabetes classification, and disease grade could not be analyzed. In the analysis, the baseline conditions should be unified in various studies and the influence of age, sex, and other factors should be corrected. Finally, there may be an optimal treatment for skin ulcers of different types and grades, but we did not classify and analyze the different grades of skin ulcers.

To sum up, the results of this meta-analysis show that compared with the basic treatment alone, the combination of Kangfuxin Liquid and basic treatment can obtain more ideal effect in the treatment of diabetic skin ulcers, improve the overall effective rate, shorten the wound rehabilitation time and hospitalization time, and have strong clinical application value.

## Figures and Tables

**Table 1:** The basic characteristics of inclusion in the literature.

Study	Year	Sample time (year.month)	Cases treat/Con	Age (years)		Sex ratio (male/female)		Course of disease (years)		Study design	Outcome measures
				Treatment group	Control group	Treatment group	Control group	Treatment group	Control group		
Ling [5]	2020	2018.8∼2019.7	40/40	72.51 ± 1.72	72.45 ± 1.63	25/15	24/16	6.71 ± 1.25	6.68 ± 1.23	RCT	① + ② + ③
Yu-zhi et al. [14]	2010	2007.8∼2008.10	42/40	49 ± 17	48 ± 16	24/18	24/16	7.2 ± 5.1	7.11 ± 4.9	RCT	① + ②
Zhiguo [15]	2019	2018.8∼2019.8	36/36	72.2 ± 8.1	71.6 ± 7.5	20/16	22/14	2.1 ± 0.1	2.3 ± 0.4	RCT	① + ② + ③
Jian-zhong et al. [16]	2018	2014.2∼2018.2	48/48	72.4 ± 8.1	73.3 ± 8.7	27/21	27/21	NR	NR	RCT	① + ② + ③
Congxiang [17]	2014	2010.11∼2012.11	30/30	62.6 ± 44.3	62.3 ± 19.8	20/10	18/12	12.2 ± 4.5	12.0 ± 4.7	RCT	①
Jian et al. [18]	2016	2014.6∼2016.6	60/60	60.78 ± 10.15	61.25 ± 10.02	31/29	30/30	NR	NR	RCT	①
Wenyao et al. [19]	2019	2018.2∼2019.6	30/30	65.33 ± 9.58	64.40 ± 11.21	17/13	18/12	14.20 ± 5.85	13.50 ± 5.53	RCT	① + ② + ③
Xiao-yan [20]	2014	2007∼2010	50/50	NR	NR	28/22	26/24	NR	NR	RCT	① + ②
Honglin and Anyuan [21]	2019	2016.5∼2017.9	50/50	50.40 ± 1.92	50.14 ± 1.53	29/21	30/20	10.87 ± 3.64	11.37 ± 3.58	RCT	① + ② + ③
Xiangjiao [22]	2017	2014.2∼2015.10	21/20	56.7 ± 9.8	55.1 ± 10.7	13/8	11/9	5.1 ± 1.9	5.1 ± 1.9	RCT	① + ②
Wenpeng et al. [23]	2011	NR	33/30	29∼50	29∼50	NR	NR	1.5∼26	1.5∼26	RCT	① + ②

*Note*. Treat: treatment; Con: control; RCT: randomized controlled trial; NR: not reported; ①: total effective rate; ②: cure time; ③: length of stay.

## Data Availability

All relevant data are presented within the manuscript.
